# Bioinformatic Resistome Profiling of Metal Tolerance Mechanisms in Endodontic Infections: Implications for Antimicrobial Nanoparticle-Based Biomaterials

**DOI:** 10.3390/jfb17050237

**Published:** 2026-05-08

**Authors:** Carlos Alberto Luna-Lara, Carlos Roberto Luna-Dominguez, Rogelio Oliver-Parra, Omaika Victoria Criollo-Barrios, María de los Dolores Vaca-Jasso, Marco Felipe Salas-Orozco

**Affiliations:** 1 Faculty of Dentistry, Autonomous University of Tamaulipas, Av. Universidad Esq. Con Blvd. Adolfo López Mateos S/N, Tampico C.P. 89337, Tamaulipas, Mexico; cluna@docentes.uat.edu.mx (C.A.L.-L.); cldominguez@uat.edu.mx (C.R.L.-D.); roliver@docentes.uat.edu.mx (R.O.-P.); omaika.criollo@uat.edu.mx (O.V.C.-B.); 2 Faculty of Stomatology, Autonomous University of San Luis Potosí, Av. Dr. Manuel Nava No. 2, Zona Universitaria, San Luis Potosí C.P. 78290, San Luis Potosí, Mexico; mdvj@uaslp.mx

**Keywords:** endodontic infections, metallic nanoparticles, metal oxide nanoparticles, efflux pumps, resistome, bioinformatics

## Abstract

Background: Metallic and metal oxide nanoparticles are increasingly explored as antimicrobial biomaterials in endodontics due to their multi-target mechanisms of action, largely mediated by metal ion release (e.g., Ag^+^, Cu^+^). However, bacterial metal resistance systems, particularly efflux-related proteins, may influence their antimicrobial performance. This study aimed to analyze the prevalence and distribution of metal resistance-associated proteins in bacteria involved in endodontic infections using a bioinformatic approach. Methods: An in silico, cross-sectional bioinformatic analysis was conducted using publicly available genomes from the Bacterial and Viral Bioinformatics Resource Center (BV-BRC). Bacterial species associated with acute apical abscess (AAA), symptomatic apical periodontitis (SAP), asymptomatic apical periodontitis (AAP), and post-treatment apical periodontitis (PTAP) were included. The presence of selected metal resistance-related proteins (CutC, CopA, CzcA, CusA, SilA, P-type ATPase, and PA3920) was assessed using a binary presence/absence framework. Prevalence, group comparisons (Fisher’s exact test), and co-occurrence patterns (Phi coefficient) were analyzed. Results: Metal resistance-associated proteins were widely distributed across all infection types, with prevalence ranging from 70.0% to 82.9% and no significant differences between groups (*p* > 0.05). CutC was the most prevalent protein, followed by CopA and CzcA, whereas SilA and PA3920 were not detected. Correlation analysis revealed consistent co-occurrence patterns among key taxa, including *Porphyromonas gingivalis*, *Fusobacterium nucleatum*, and *Prevotella* spp. Conclusions: Metal resistance-related proteins are broadly distributed in endodontic microbiota, indicating a conserved genetic capacity for metal tolerance. These findings suggest that microbial resistance determinants may influence, but do not directly determine, the antimicrobial performance of nanoparticle-based biomaterials. This study provides a hypothesis-generating, bioinformatic framework to support the design and optimization of antimicrobial biomaterials, highlighting the need for experimental validation and integration of phenotypic and biofilm-based analyses.

## 1. Introduction

Endodontic infections continue to represent a significant clinical challenge due to the complex architecture of the root canal system and the ability of microorganisms to persist within inaccessible niches, forming highly organized biofilms that are resistant to conventional disinfection protocols. Despite advances in instrumentation, irrigation, and obturation techniques, therapeutic failure remains closely associated with the survival of resistant microorganisms, particularly species such as *Enterococcus faecalis*, which are recognized for their ability to adapt to harsh environments and for their involvement in persistent infections and post-treatment disease [1,2].

In this context, metallic nanoparticles have emerged as an innovative antimicrobial alternative in dentistry, particularly in endodontics, owing to their unique physicochemical properties, including nanoscale size, high surface area-to-volume ratio, and increased chemical reactivity. These characteristics confer multiple mechanisms of action, such as the generation of reactive oxygen species, disruption of bacterial membrane integrity, interaction with intracellular proteins, and DNA damage, collectively reducing the likelihood of resistance development compared with conventional antimicrobials [3,4,5]. Importantly, a substantial proportion of the antimicrobial activity of metallic nanoparticles is mediated through the release of metal ions (e.g., Ag^+^, Cu^+^, Zn^2+^), which interact with key cellular targets such as thiol groups in proteins, enzymes involved in respiration, and nucleic acids, ultimately leading to bacterial cell death. In particular, silver-, zinc-, copper-, and titanium-based nanoparticles have demonstrated effectiveness against both Gram-positive and Gram-negative microorganisms, as well as against complex biofilms associated with endodontic infections [6].

However, increasing evidence suggests that bacterial adaptive mechanisms originally evolved for survival under metal stress may also influence the antimicrobial performance of nanoparticle-based biomaterials. In particular, bacterial metal resistance systems—including efflux pumps, metal-binding proteins, and homeostasis regulators—are capable of recognizing, transporting, and detoxifying metal ions released from nanoparticles. Efflux systems such as P-type ATPases (e.g., CopA), cation diffusion facilitators (CDF), and RND-type transporters (e.g., CzcA, CusA) can actively extrude toxic ions from the cytoplasm, thereby reducing intracellular accumulation and mitigating cellular damage. This establishes a direct mechanistic link between classical metal resistance pathways and the biological response to nanoparticle-derived antimicrobial stress [7,8]. From a biomaterials perspective, this interaction is highly relevant. The effectiveness of antimicrobial nanoparticles depends not only on their physicochemical properties but also on the ability of target microorganisms to tolerate or counteract metal-induced toxicity. Consequently, the presence of metal resistance systems may represent an intrinsic biological factor that modulates the functional performance of nanoparticle-based biomaterials in clinical environments. Furthermore, these systems are frequently associated with cross-resistance phenomena involving antibiotics and biocides. This highlights their role in broader multidrug resistance networks [9].

From a clinical and microbiological perspective, the existence of metal resistance systems represents a concerning scenario, particularly in endodontic infections. In these settings, selective pressure derived from the use of irrigants, intracanal medicaments, and restorative materials may promote the selection of multidrug-resistant phenotypes. Therefore, understanding the distribution of these resistance systems is essential not only for microbiological characterization but also for the rational design and optimization of next-generation antimicrobial biomaterials [10].

The advent of bioinformatics and access to large-scale genomic databases have enabled the systematic exploration of the distribution and prevalence of genes and proteins related to antimicrobial resistance across multiple bacterial species. Platforms such as the Bacterial and Viral Bioinformatics Resource Center (BV-BRC) facilitate the integration of genomic data, functional annotations, and analytical tools. These platforms enable robust comparative studies that contribute to the identification of resistance patterns and the characterization of the resistome in different clinical contexts. Such approaches provide valuable insights into the resistome landscape. These insights may influence biomaterial performance prior to experimental validation [11].

Despite the growing interest in the application of nanoparticles in dentistry, there remains limited understanding regarding the prevalence and distribution of proteins associated with resistance to metallic nanoparticles in bacteria involved in endodontic infections. There is also limited knowledge about their potential relationship with different types of periapical pathology. This knowledge gap limits the development of rational therapeutic strategies that integrate nanomaterials with approaches aimed at counteracting bacterial resistance mechanisms [10].

Therefore, the aim of the present study is to analyze the prevalence of proteins associated with resistance to metallic nanoparticles in bacteria involved in different types of endodontic infections using a bioinformatic approach based on publicly available genomic databases. By characterizing the resistome associated with metal tolerance, this study seeks to provide a biologically informed framework to better understand potential microbial factors influencing the functional performance of metallic nanoparticle-based biomaterials. It also aims to contribute to the development of more effective antimicrobial strategies in endodontics.

## 2. Materials and Methods

### 2.1. Study Design

This study was designed as an in silico, cross-sectional bioinformatic analysis to evaluate the prevalence and co-occurrence patterns of proteins associated with resistance to metallic and metal oxide nanoparticles in bacteria implicated in endodontic infections.

### 2.2. Selection of Bacterial Species

Bacterial taxa associated with endodontic infections were identified based on the twelfth edition of Pathways of the Pulp. Four clinical conditions were considered: acute apical abscess (AAA), symptomatic apical periodontitis (SAP), asymptomatic apical periodontitis (AAP), and post-treatment apical periodontitis (PTAP). All bacterial species reported for each condition were compiled and categorized into their respective groups [12].

### 2.3. Genomic Data Source and Retrieval

Genomic data were obtained from the Bacterial and Viral Bioinformatics Resource Center (BV-BRC), an integrated platform that aggregates publicly available bacterial genomes, primarily from the National Center for Biotechnology Information (NCBI). BV-BRC provides standardized genome annotation using the Rapid Annotation using Subsystem Technology toolkit (RASTtk), ensuring consistent identification of coding sequences, protein functions, virulence factors, and antimicrobial resistance determinants. Each bacterial species was individually queried within the BV-BRC database as part of the data retrieval process. Species without available genomic data were recorded as not available (N/A) and excluded from subsequent analyses [11]. Given that BV-BRC contains multiple genomes per species, the analysis was conducted at the species level. Available genomes were evaluated collectively to determine the presence of resistance-associated proteins. This approach does not rely on a single reference genome. Instead, it reflects the potential functional capacity of each species based on available genomic evidence.

### 2.4. Genome Inclusion Criteria

To ensure biological relevance and data quality, genomes were filtered according to the following criteria:

- Genome status: Complete genome or whole genome shotgun (WGS)
- Genome quality: “Good” (as defined by BV-BRC quality metrics)
- Host group: Human

Genomes derived from non-human hosts were excluded to better reflect clinically relevant oral isolates. Only genomes meeting all criteria were included in the analysis.

### 2.5. Protein Selection and Annotation Strategy

A targeted protein-based approach was used to identify determinants associated with resistance to metallic nanoparticles. The selected efflux-related proteins were chosen because they have been previously reported to participate in bacterial resistance to metal-based nanoparticles, particularly through mechanisms involving metal ion extrusion, detoxification, and homeostasis [13]:

- CutC (copper homeostasis protein)
- CopA (P-type ATPase copper efflux transporter)
- CzcA (cation efflux system protein; Zn/Cd/Co resistance)
- CusA (copper/silver efflux transporter)
- SilA (silver efflux system component)
- P-type heavy metal translocating ATPase
- PA3920 (metal-translocating ATPase homolog)

Protein presence was determined using the BV-BRC protein annotation interface. Manual curation was performed by querying protein names and functional annotations within each genome. Only annotated proteins with functional assignment consistent with resistance-related activity were considered.

### 2.6. Data Curation and Binary Matrix Construction

For each bacterial species, the presence (1) or absence (0) of each target protein was recorded, generating a binary dataset. Separate presence/absence matrices were constructed for each infection category. All datasets were curated and organized using Microsoft Excel version 2024 (Microsoft Corp., Redmond, WA, USA) and subsequently exported for statistical analysis. This binary framework reflects genomic presence or absence of annotated proteins, and therefore represents the potential capacity of each species to harbor resistance-associated systems, rather than direct evidence of gene expression, protein activity, or phenotypic resistance under clinical or experimental conditions. Additionally, this approach does not account for gene copy number, allelic variation, or operon structure, which may influence functional resistance but are beyond the scope of this analysis.

### 2.7. Statistical Analysis

All statistical analyses were performed using a binary presence/absence framework derived from the resistome matrix, where each bacterial species was evaluated for the presence of resistance-associated proteins across different endodontic infection types.

Descriptive analyses were initially conducted to characterize the distribution of resistance-associated proteins. The number of proteins per bacterial species was quantified and visualized using bar plots, allowing comparison of resistome structure across infection types. Additionally, the proportion of bacterial species harboring at least one resistance-associated protein was calculated for each clinical condition (AAA, SAP, AAP, and PTAP), providing an overall measure of resistome prevalence.

Prevalence analysis was performed for each protein at both global and infection-specific levels. The prevalence was calculated as the proportion of bacterial species in which each protein was detected. Corresponding 95% confidence intervals (95% CIs) were estimated using binomial exact methods to account for the binary nature of the data and relatively small sample sizes.

To compare the overall presence of resistance-associated proteins between infection types, contingency tables were constructed based on the number of species with at least one detected protein versus those without detectable proteins. Fisher’s exact test was applied to evaluate statistical differences between groups due to the categorical nature of the data and expected small cell counts. Odds ratios (ORs) were calculated using acute apical abscess (AAA) as the reference category to estimate the relative likelihood of protein presence across infection types.

Correlation analyses were performed to evaluate co-occurrence patterns of resistance-associated proteins among bacterial species within each infection type. Binary matrices were constructed and pairwise associations between bacterial species were quantified using the Phi (φ) coefficient, a correlation measure appropriate for dichotomous variables. The resulting correlation matrices were visualized as heatmaps to identify clustering patterns and similarities in resistome profiles across taxa. Bacterial species were treated as analytical units. Therefore, statistical were interpreted as exploratory patterns of resistome distribution rather than strictly independent observations. Similarly, Fisher’s exact test provides comparative prevalence estimates without implying biological causality, and Phi coefficients reflect co-occurrence patterns rather than direct biological interactions.

**Figure 1:**
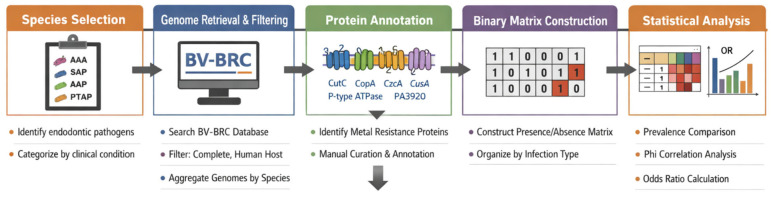
Workflow of the bioinformatic analysis pipeline for resistance-associated proteins in endodontic infections. Species selection (AAA, SAP, AAP, PTAP), genome retrieval/filtering (BV-BRC), protein annotation, and binary matrix construction were performed, followed by statistical analyses (prevalence, Phi correlation, and odds ratios).

All analyses were conducted using Python (3.14.x) (pandas (3.0.2), NumPy(2.4.0), and Matplotlib (3.10.9) libraries), ensuring reproducibility and standardized data processing. A significance level of *p* < 0.05 was established for all inferential tests (Figure 1).

## 3. Results

### 3.1. Distribution of Resistance-Associated Proteins Across Bacterial Species

**Figure 2:**
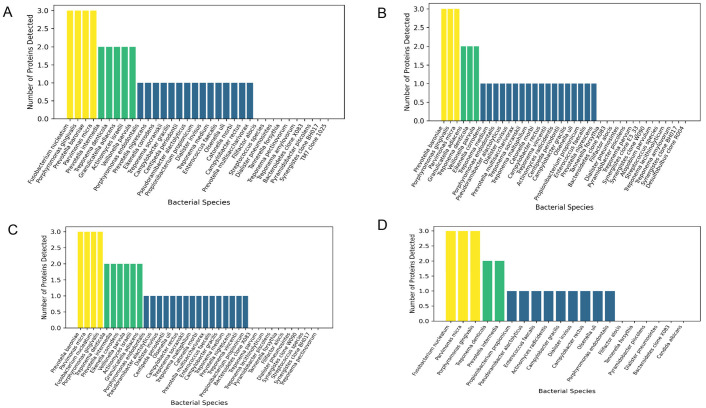
Distribution of resistance-associated proteins across bacterial species in (**A**) acute apical abscess (AAA), (**B**) symptomatic apical periodontitis (SAP), (**C**) asymptomatic apical periodontitis (AAP), and (**D**) post-treatment apical periodontitis (PTAP). Bars represent the number of proteins detected per species, and color intensity reflects protein abundance.

The distribution of resistance-associated proteins across bacterial species differed according to the clinical condition (Figure 2A–D). These differences reveal distinct patterns of resistome organization.

In acute apical abscess (AAA) (Figure 2A), the resistome displayed a highly concentrated structure. A limited number of taxa harbored the highest number of proteins. *Fusobacterium nucleatum*, *Porphyromonas gingivalis*, *Parvimonas micra*, and *Prevotella baroniae* showed the greatest protein counts (three proteins each). A secondary group exhibited intermediate values (two proteins), whereas the majority showed low frequencies (≤1 protein). Overall, 79.4% of bacterial species presented at least one resistance-associated protein, while 20.6% showed no detectable proteins. This indicates a predominantly protein-positive microbial profile.

In symptomatic apical periodontitis (SAP) (Figure 2B), the resistome exhibited an intermediate organization, with *Prevotella baroniae*, *Porphyromonas gingivalis*, and *Parvimonas micra* again presenting the highest protein counts (three proteins). Most remaining taxa showed low-level presence. In this condition, 76.3% of bacterial species harbored resistance-associated proteins, whereas 23.7% showed no detectable proteins, reflecting a transitional distribution.

In asymptomatic apical periodontitis (AAP) (Figure 2C), the resistome demonstrated a more distributed configuration, with a greater number of taxa exhibiting intermediate protein counts. Although several species reached the highest values (three proteins), the distribution was more homogeneous. In this group, 82.9% of bacterial species presented resistance-associated proteins, while 17.1% lacked detectable proteins, indicating the highest proportion of protein-positive taxa among all conditions.

In contrast, post-treatment apical periodontitis (PTAP) (Figure 2D) exhibited a re-concentrated resistome pattern, with a reduced subset of taxa accumulating the highest number of proteins. In this condition, 70.0% of bacterial species presented resistance-associated proteins, whereas 30.0% showed no detectable proteins, representing the lowest proportion of protein-positive taxa.

Importantly, a subset of bacterial species consistently exhibited high protein counts across multiple infection types, indicating a stable association with resistance-related functions. *Porphyromonas gingivalis* and *Parvimonas micra* ranked among the top species (three proteins) in all conditions (AAA, SAP, AAP, and PTAP). *Fusobacterium nucleatum* and *Prevotella baroniae* also showed recurrent high protein counts in multiple conditions, while *Treponema denticola* consistently exhibited intermediate protein levels (two proteins). Additionally, *Granulicatella adiacens* and *Prevotella intermedia* displayed repeated intermediate profiles.

In Table 1, the prevalence analysis demonstrated a heterogeneous and non-core resistome across endodontic infections, with cutC as the most prevalent protein globally (58.7%) and consistently high across all infection types, indicating a dominant role of copper-associated resistance mechanisms. CopA and CzcA showed moderate and stable prevalence, suggesting a secondary and context-dependent contribution to metal homeostasis, while CusA and P-type ATPases were infrequent and variably distributed. Notably, SilA and PA3920 were completely absent, indicating a lack of silver-associated resistance systems. Overall, these findings highlight a resistome primarily driven by copper tolerance, with a modular distribution of additional efflux components and absence of silver-related determinants.

### 3.2. Correlation Analysis Across Endodontic Infections

**Figure 3:**
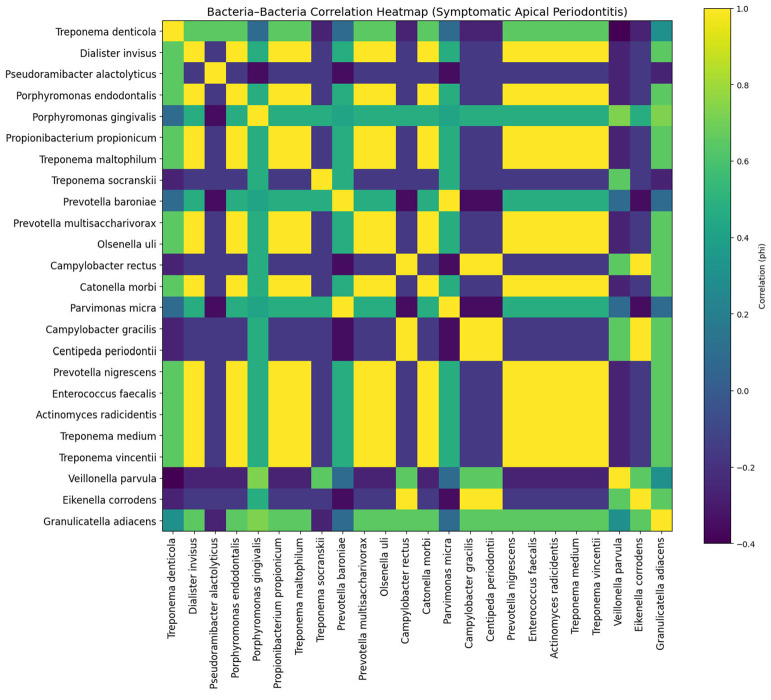
Bacteria–bacteria correlation heatmap (φ coefficient) in acute apical abscess (AAA), illustrating patterns of co-occurrence of resistance-associated proteins among bacterial species.

In acute apical abscess (AAA), the correlation structure revealed a well-defined cluster of bacteria sharing similar resistance-associated protein profiles, particularly centered around *Porphyromonas gingivalis*, *Fusobacterium nucleatum*, *Prevotella intermedia*, and *Veillonella parvula*. These species showed moderate-to-strong positive correlations, suggesting coordinated resistome patterns during acute infection. Additional associations were observed among *Granulicatella adiacens* and *Actinomyces israelii*, indicating secondary clustering. In contrast, species such as *Treponema socranskii* and *Campylobacter gracilis* displayed weaker or negative correlations, reflecting more independent resistome profiles (Figure 3).

**Figure 4:**
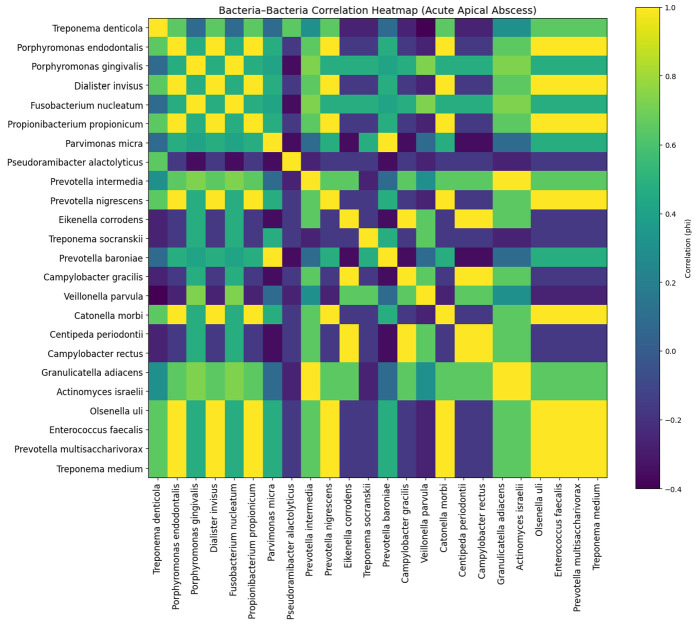
Bacteria–bacteria correlation heatmap (φ coefficient) in symptomatic apical periodontitis (SAP), showing variability in co-occurrence patterns of resistance-associated proteins across bacterial species.

In symptomatic apical periodontitis (SAP), the correlation matrix exhibited a more heterogeneous and modular organization. While the core cluster (*P. gingivalis*, *F. nucleatum*, *Prevotella* spp.) remained partially conserved, correlations were less uniform and more fragmented. Certain species, including *Parvimonas micra* and *Prevotella baroniae*, showed selective associations, whereas others such as *Treponema* spp. demonstrated variable connectivity. This pattern suggests a transitional or less stabilized resistome structure compared to acute infections (Figure 4).

**Figure 5:**
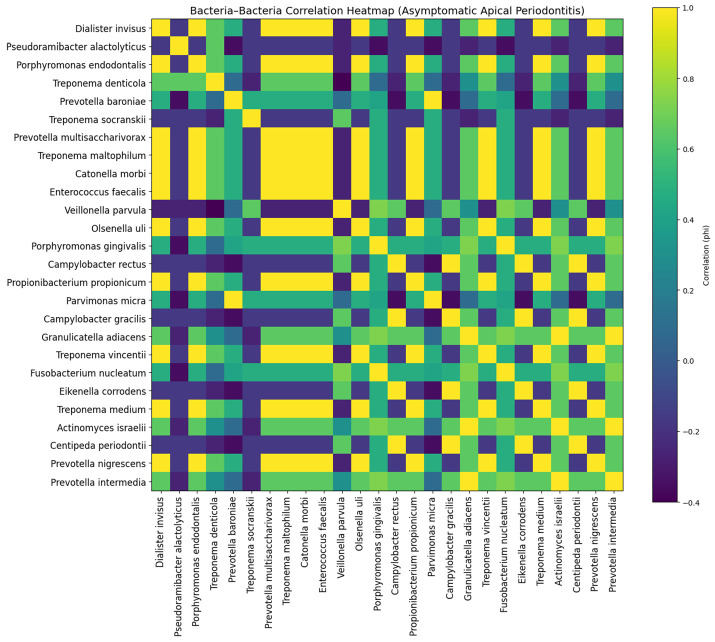
Bacteria–bacteria correlation heatmap (φ coefficient) in asymptomatic apical periodontitis (AAP), displaying consistent co-occurrence patterns of resistance-associated proteins among bacterial species.

In asymptomatic apical periodontitis (AAP), a more consolidated and stable clustering pattern was observed. Multiple bacterial species, including *P. gingivalis*, *F. nucleatum*, *Prevotella intermedia*, *Actinomyces israelii*, and *Granulicatella adiacens*, exhibited strong positive correlations, indicating a shared and persistent resistome configuration. The presence of widespread positive associations suggests that chronic asymptomatic infections may harbor a more uniform and co-adapted microbial community. Conversely, a limited number of species maintained weak or neutral correlations, reflecting minor ecological divergence (Figure 5).

**Figure 6:**
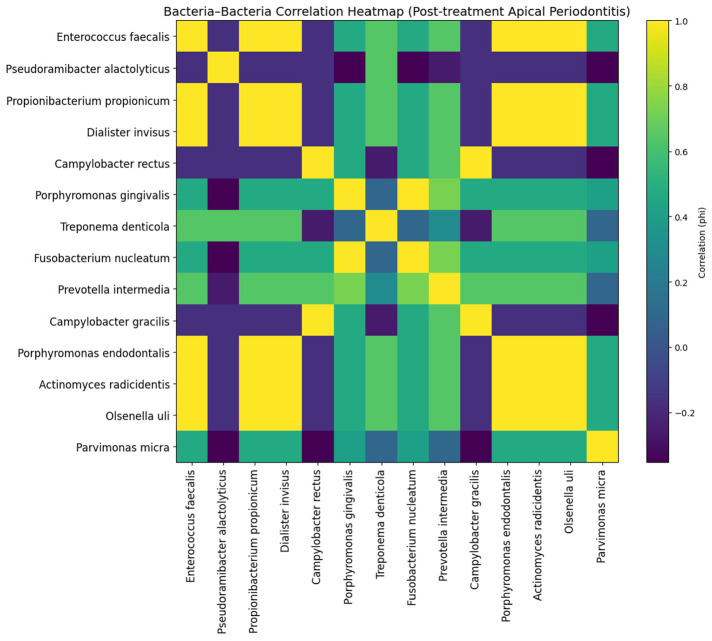
Bacteria–bacteria correlation heatmap (φ coefficient) in post-treatment apical periodontitis (PTAP), illustrating patterns of co-occurrence of resistance-associated proteins across bacterial species.

In post-treatment apical periodontitis (PTAP), the correlation structure appeared more compact and reinforced, with fewer but stronger associations. A dominant cluster involving *P. gingivalis*, *F. nucleatum*, *Prevotella intermedia*, and *Treponema denticola* persisted, displaying high correlation values indicative of strong co-selection. Notably, *Enterococcus faecalis* showed broader but less specific correlations, consistent with a generalist resistance profile. Some taxa, such as *Campylobacter* spp., exhibited reduced connectivity, suggesting selective filtering of the microbial community following treatment. Overall, PTAP demonstrated the most constrained and selection-driven resistome architecture among the four infection types (Figure 6).

### 3.3. Comparison of Overall Prevalence of Resistance-Associated Proteins Across Infection Types

In Table 2, the comparative analysis of the overall prevalence of resistance-associated proteins across endodontic infection types revealed a consistently high presence in all groups, ranging from 70.0% to 82.9%. Asymptomatic apical periodontitis (AAP) exhibited the highest prevalence (82.9%), followed by acute apical abscess (AAA) (79.4%) and symptomatic apical periodontitis (SAP) (76.3%), while post-treatment apical periodontitis (PTAP) showed the lowest prevalence (70.0%). Despite these differences, no statistically significant associations were observed between infection type and the presence of resistance-related proteins (*p* > 0.05). Odds ratio analysis indicated a non-significant trend toward higher prevalence in AAP (OR = 1.26) and lower prevalence in PTAP (OR = 0.60) compared to the reference group (AAA). Overall, these findings suggest that resistance-associated proteins are widely distributed across endodontic infections, with only minor, non-significant variations among clinical conditions.

## 4. Discussion

This bioinformatic study evaluated the prevalence and distribution of efflux pump-related proteins associated with resistance to metallic nanoparticles across four endodontic infection types: acute apical abscess (AAA), symptomatic apical periodontitis (SAP), asymptomatic apical periodontitis (AAP), and post-treatment apical periodontitis (PTAP). The proteins analyzed included CutC, CopA, CzcA, CusA, the heavy metal–translocating P-type ATPase, SilA, and PA3920 [14]. This study should be interpreted within the context of biomaterials research. It provides a biologically informed framework to understand how microbial resistance determinants may influence the functional performance of metallic nanoparticle-based antimicrobial systems.

A high prevalence of resistance-associated proteins was observed across all infection types (70.0–82.9%), with no statistically significant differences between groups (*p* > 0.05). This finding suggests that efflux-mediated resistance mechanisms are broadly distributed within endodontic microbiota, independent of infection type or clinical presentation. These results are consistent with previous reports demonstrating a high prevalence of nanoparticle resistance-related genes in endodontic infections. Clinically, these results raise concerns regarding the effectiveness of metal-based antimicrobial strategies, including the use of metallic nanoparticles as adjuncts in root canal disinfection, as efflux-mediated mechanisms may contribute to reduced susceptibility rather than definitively limiting antimicrobial efficacy. This highlights the need for combined or alternative disinfection approaches capable of overcoming bacterial resistance. It also underscores the importance of optimizing irrigation protocols and intracanal medicaments to improve treatment outcomes in both primary and secondary endodontic infections [7].

It is important to note that this binary framework reflects the genomic presence or absence of annotated proteins. Therefore, it represents the potential capacity of each species to harbor resistance-associated systems, rather than direct evidence of gene expression, protein activity, or phenotypic resistance under clinical or experimental conditions. Accordingly, the resistome data presented here reflect potential resistance traits and should not be interpreted as confirmed functional resistance to metallic nanoparticles.

Among the proteins analyzed, CutC was the most prevalent across all infection types, followed by CopA, whereas SilA and PA3920 were not detected. CutC is involved in copper homeostasis and contributes to intracellular metal regulation. This supports bacterial survival under metal-induced stress [15]. Similarly, CopA and CzcA have been implicated in the active efflux of toxic metal ions. This reinforces their role in resistance to metal-based antimicrobials [16,17]. These proteins should be interpreted as general metal resistance systems with indirect relevance to nanoparticles, primarily due to the fact that many metallic nanoparticles exert antimicrobial activity through ion release (e.g., Ag^+^, Cu^+^). Importantly, copper efflux systems are mechanistically linked to resistance to silver nanoparticles. Both copper (Cu^+^) and silver (Ag^+^) ions share similar physicochemical properties, including ionic radius and coordination chemistry. This allows them to interact with the same cellular targets, such as thiol groups in proteins. As a result, transporters such as CopA and CzcCBA can recognize and expel silver ions in addition to copper. This contributes to cross-resistance between these metals [18]. These systems therefore represent key bacterial defense mechanisms that may influence, but do not directly determine, the antimicrobial performance of metallic nanoparticle-based biomaterials [19].

At the microbial level, *Fusobacterium nucleatum*, *Porphyromonas gingivalis*, *Parvimonas micra*, and *Prevotella baroniae* consistently exhibited higher frequencies of resistance-associated proteins. These species are known to interact synergistically within polymicrobial biofilms. This interaction enhances their persistence and pathogenicity in endodontic infections. Their co-occurrence may also facilitate the maintenance and dissemination of resistance traits, particularly in complex biofilm environments. From a biomaterials perspective, these structured microbial communities may significantly influence nanoparticle performance, as biofilm-associated bacteria often exhibit reduced susceptibility to antimicrobial agents due to limited penetration, altered metabolic states, and cooperative resistance mechanisms [20,21].

The oral cavity has been recognized as an important reservoir of antimicrobial resistance genes (ARGs), with horizontal gene transfer playing a key role in their dissemination [22]. This phenomenon may extend to resistance against metallic nanoparticles. It supports a potential co-selection between antibiotic resistance and metal resistance mechanisms. Indeed, previous studies have demonstrated the coexistence of ARGs and metal resistance genes in oral biofilms. These findings highlight the importance of incorporating resistance-aware strategies in the design of antimicrobial biomaterials, particularly to minimize the risk of co-selection and persistence of resistant phenotypes [23].

Although *Enterococcus faecalis* did not show the highest number of resistance-associated proteins, it consistently exhibited their presence. This finding aligns with previous studies demonstrating its capacity to develop adaptive resistance to silver nanoparticles through the upregulation of efflux systems such as CopA and CzcCBA. These observations further support the need to consider microbial adaptive potential when designing nanoparticle-based therapeutic strategies in endodontics [24,25].

A limitation of the present study is that the bacterial dataset was derived from human-associated isolates rather than exclusively from endodontic infection-specific samples. This may introduce potential bias, as the analyzed genomes may not fully represent the microbial composition and resistome of infection-specific endodontic microbiomes. This approach was justified by the limited availability of well-annotated genomic and metagenomic datasets directly obtained from endodontic infections. Such studies remain relatively scarce and technically demanding due to sampling constraints and the complexity of polymicrobial biofilms. Indeed, metagenomic and metatranscriptomic approaches in endodontics are still emerging. They have only recently begun to characterize the microbial composition and resistome of these infections [26].

Moreover, microbiome studies have demonstrated that a significant proportion of microbial diversity cannot be fully captured. This limitation is due to constraints in sequencing depth, incomplete reference databases, and the presence of uncultivable or low-abundance taxa. Therefore, while the inclusion of human-associated genomes may limit direct clinical extrapolation, it provides a broader ecological representation of bacterial taxa with potential relevance to endodontic infections, supported by data availability and annotation quality. Consequently, the inclusion of human-derived bacterial species commonly associated with oral and endodontic microbiota allowed for a more comprehensive and statistically robust analysis of resistance-associated proteins. Importantly, many of the selected taxa are well-recognized members of endodontic infections. This supports the biological relevance of this approach. Nonetheless, future studies should prioritize the generation and integration of high-quality, infection-specific multi-omics datasets to further validate and refine these findings [27].

From a translational biomaterials perspective, bioinformatic screening of resistome profiles represents a valuable preliminary step in anticipating potential microbial responses to nanoparticle-based therapies. These approaches can guide the rational design and optimization of biomaterials by identifying resistance-associated targets that may compromise antimicrobial performance. In this context, future research should focus on: (i) experimental validation of bacterial susceptibility to specific metallic nanoparticles [28,29], (ii) integration of genomic resistome data with phenotypic resistance assays [30,31], and (iii) evaluation of nanoparticle efficacy in biofilm-based models that better reflect clinical conditions [32,33]. Additionally, resistance-aware biomaterial design strategies—such as controlled ion release systems, surface functionalization, and combination therapies with efflux pump inhibitors—should be explored to enhance antimicrobial effectiveness and overcome microbial adaptive responses.

## 5. Conclusions

Efflux pump-related proteins associated with resistance to metallic nanoparticles are highly prevalent across endodontic infections, with no significant differences between infection types, suggesting that efflux-mediated mechanisms are a conserved feature of the endodontic microbiota. However, these findings should be interpreted as reflecting the potential presence of resistance-associated systems rather than confirmed phenotypic resistance to nanoparticles. In this context, such mechanisms may influence, but do not directly determine, the antimicrobial performance of nanoparticle-based biomaterials. This study should therefore be considered hypothesis-generating. It provides a bioinformatic framework to identify microbial factors relevant to biomaterials development. From a translational perspective, these results highlight the importance of incorporating resistance profiles into the design and optimization of antimicrobial biomaterials. Future studies integrating experimental validation with nanoparticles, resistome–phenotype correlation, and biofilm-based models will be essential to confirm their functional implications in endodontic applications.

## Figures and Tables

**Table 1:** Prevalence of efflux pump-related proteins across endodontic infections.

Protein	Global % (95% CI)	Abscess % (95% CI)	Symptomatic AP % (95% CI)	Asymptomatic AP % (95% CI)	Post-Treatment % (95% CI)
cutC	58.7 (48.5–68.2)	65.5 (48.3–79.6)	53.3 (34.3–71.7)	56.3 (37.7–73.6)	61.1 (38.6–79.7)
CopA	29.4 (21.1–39.3)	34.5 (20.5–51.7)	23.3 (10.9–42.1)	31.3 (16.1–51.4)	27.8 (12.5–50.9)
CzcA	18.3 (11.7–27.5)	20.7 (10.0–38.4)	16.7 (6.0–36.5)	18.8 (7.7–37.2)	16.7 (5.1–42.0)
CusA	6.4 (3.2–12.5)	6.9 (2.0–21.7)	6.7 (1.2–29.8)	6.3 (1.1–28.3)	5.6 (1.0–25.8)
SilA	0.0 (0.0–3.8)	0.0 (0.0–11.7)	0.0 (0.0–13.8)	0.0 (0.0–13.2)	0.0 (0.0–16.8)
PA3920	0.0 (0.0–3.8)	0.0 (0.0–11.7)	0.0 (0.0–13.8)	0.0 (0.0–13.2)	0.0 (0.0–16.8)

**Table 2:** Comparison of overall prevalence of resistance-associated proteins across endodontic infection types.

Infection Type	Presence (%)	Absence (%)	Comparison vs. Others (OR)	*p*-Value (Fisher’s Exact Test)
Acute apical abscess (AAA)	79.4	20.6	Reference	—
Symptomatic apical periodontitis (SAP)	76.3	23.7	0.83	>0.05
Asymptomatic apical periodontitis (AAP)	82.9	17.1	1.26	>0.05
Post-treatment apical periodontitis (PTAP)	70.0	30.0	0.60	>0.05

## Data Availability

The datasets analyzed in this study are publicly available in the Bacterial and Viral Bioinformatics Resource Center (BV-BRC). The list of bacterial taxa, genome identifiers/accession numbers, and the presence/absence matrices used for the analyses should be made available by the corresponding author upon reasonable request.
